# Impact of Variant Allele Frequency (VAF) Levels on Clinical Efficacy of Osimertinib in Patients with Metastatic NSCLC

**DOI:** 10.3390/medsci14020233

**Published:** 2026-05-01

**Authors:** Abed Agbarya, Kamel Mhameed, Arina Soklakova, Haitam Nasrallah, Mahmoud Abu Amna, Sabri El-Saied, Mohammad Sheikh-Ahmad, Walid Shalata

**Affiliations:** 1 The Ruth and Bruce Rappaport Faculty of Medicine, Technion-Israel Institute of Technology, Haifa 3109601, Israel; abed.agbarya@b-zion.org.il (A.A.); mahmud_ab@clalit.org.il (M.A.A.); 2 Department of Oncology, Bnai-Zion Medical Center, Haifa 3339419, Israel; 3 The Joseph Fishman Oncology Center, Rambam Medical Center, Haifa 3525409, Israel; k_mhameed@rmc.gov.il (K.M.);; 4 Medical School for International Health, Faculty of Health Sciences, Ben Gurion University of the Negev, Beer Sheva 8410501, Israel; 5 Faculty of Health Sciences, Ben Gurion University of the Negev, Ben Gurion Avenue, Beer Sheva 8410501, Israel; 6 The Legacy Heritage Cancer Center, Dr. Larry Norton Institute, Soroka Medical Center, Beer Sheva 8410501, Israel

**Keywords:** non-small cell lung cancer (NSCLC), epidermal growth factor receptor (EGFR), variant allele frequency (VAF), circulating tumor DNA (ctDNA), liquid biopsy, EGFR tyrosine kinase inhibitor (EGFR TKI), osimertinib

## Abstract

**Background**: Non-small cell lung cancer (NSCLC) remains the leading cause of cancer-related mortality despite major advances in diagnostics and therapies. The prognosis remains poor, mostly due to late-stage presentation and molecular heterogeneity. Epidermal growth factor receptor (EGFR) mutations are common drivers of NSCLC. The development of EGFR tyrosine kinase inhibitors (TKIs) has significantly improved outcomes in patients with EGFR mutations. Variant allele frequency (VAF) is a quantitative genomic measure representing the proportion of sequencing reads harboring a given mutation. In NSCLC tissue, the EGFR mutation VAF reflects tumor clonality and intratumoral heterogeneity, and accumulating evidence suggests an association between EGFR VAF and response to EGFR-targeted TKIs. **Methods**: To address the limited synthesis of data on the relevance of EGFR mutation VAF in NSCLC, we conducted a narrative review of the literature using PubMed/MEDLINE and Embase databases and current clinical guidelines, synthesizing available evidence on EGFR VAF, including its biological, molecular, and therapeutic implications in EGFR-mutated disease. The review was structured in accordance with the SANRA (Scale for the Assessment of Narrative Review Articles) checklist. **Results**: EGFR VAF and on-treatment VAF dynamics are consistently associated with treatment response, progression-free survival, and overall survival in osimertinib-treated NSCLC. Baseline VAF enables risk stratification, early clearance kinetics predict durable benefit, and longitudinal VAF monitoring facilitates early detection of resistance. Importantly, the prognostic implications of VAF differ fundamentally between tissue-based and plasma-based measurements: high tissue VAF reflects clonal homogeneity and predicts favorable TKI response, whereas high plasma VAF indicates elevated tumor burden and is associated with inferior outcomes. In the second-line setting, the T790M/activating mutation ratio serves as a surrogate for resistance clonality and independently predicts osimertinib efficacy. **Conclusions**: EGFR VAF represents a promising dynamic molecular biomarker for treatment monitoring and precision decision-making in EGFR-mutated NSCLC.

## 1. Introduction

Lung cancer remains the leading cause of cancer-related mortality worldwide, accounting for approximately 1.8 million deaths annually [1]. Despite significant advances in systemic therapies and molecular diagnostics, the overall prognosis remains poor, largely due to late-stage presentation and pronounced biological heterogeneity. Lung cancer is broadly divided into small cell lung cancer (SCLC) and non-small cell lung cancer (NSCLC), with NSCLC representing nearly 85% of all cases. NSCLC encompasses several histological subtypes, including adenocarcinoma, squamous cell carcinoma, and large cell carcinoma, with adenocarcinoma being the most prevalent and the subtype most frequently associated with oncogenic driver mutations [2].

The molecular characterization of NSCLC has led to the identification of actionable genomic alterations that have transformed treatment paradigms. Among these, activating mutations in the epidermal growth factor receptor (EGFR) gene are among the most clinically significant. EGFR mutations occur in approximately 10–15% of NSCLC patients in Western populations and up to 40–50% in East Asian populations, with a higher prevalence among never-smokers, females, and patients with adenocarcinoma histology [3]. The most common sensitizing EGFR mutations are exon 19 deletions and the exon 21 L858R point mutation, which drive constitutive activation of downstream signaling pathways involved in tumor proliferation and survival [4].

Beyond EGFR, the molecular landscape of NSCLC encompasses a growing number of actionable driver alterations, including ALK rearrangements, ROS1 fusions, BRAF V600E mutations, KRAS G12C mutations, MET exon 14 skipping alterations, RET fusions, and NTRK fusions, each with corresponding targeted therapeutic strategies. While this review focuses specifically on EGFR-mutated disease, the principles of VAF-based molecular monitoring may ultimately prove applicable across multiple oncogenic drivers. The development of EGFR tyrosine kinase inhibitors (TKIs) has dramatically improved outcomes for patients with EGFR-mutated NSCLC. Osimertinib, a third-generation, irreversible EGFR TKI, was initially developed to target tumors harboring the EGFR T790M resistance mutation following first- or second-generation EGFR TKI therapy [5]. Subsequent clinical trials demonstrated superior efficacy of osimertinib in the first-line setting, with improved progression-free and overall survival, enhanced central nervous system penetration, and a favorable toxicity profile compared with earlier-generation TKIs [6]. As a result, osimertinib is now considered the standard first-line therapy for advanced EGFR-mutated NSCLC. Nevertheless, clinical responses to osimertinib vary substantially, and resistance inevitably develops, highlighting the need for improved predictive and prognostic biomarkers beyond the presence of EGFR mutations alone.

Variant allele frequency (VAF) has emerged as a quantitative genomic parameter that may provide additional biological and clinical insight. VAF is defined as the proportion of sequencing reads containing a specific genetic variant relative to the total number of reads at that genomic locus [7]. In tumor tissue, the EGFR mutation VAF reflects the proportion of cancer cells harboring the mutation and may serve as a surrogate marker for tumor clonality and intratumoral heterogeneity. In circulating tumor DNA (ctDNA), VAF is influenced by both tumor burden and biological shedding dynamics, allowing for non-invasive assessment and longitudinal monitoring during treatment [8].

Increasing evidence suggests that EGFR mutation VAF may be associated with treatment outcomes in patients receiving EGFR TKIs. Higher EGFR VAF has been correlated with improved response rates and longer progression-free and overall survival in tissue-based analyses, whereas elevated plasma VAF may reflect greater tumor burden and heterogeneity, potentially contributing to inferior outcomes [9]. Furthermore, changes in VAF over time, particularly in ctDNA, may provide early indications of treatment response or emerging resistance during osimertinib therapy [10]. Understanding the clinical implications of EGFR VAF is therefore critical for refining precision medicine approaches and optimizing therapeutic strategies in EGFR-mutated NSCLC. The present narrative review synthesizes the available evidence across the full clinical trajectory—from baseline risk stratification through on-treatment dynamic monitoring to resistance characterization—offering an integrated conceptual framework for the role of VAF in osimertinib-treated NSCLC.

## 2. Materials and Methods

A narrative review of the literature was conducted to synthesize the available evidence on EGFR mutation VAF and its clinical implications in NSCLC treated with osimertinib. The review was structured in accordance with the Scale for the Assessment of Narrative Review Articles (SANRA) checklist to ensure methodological transparency appropriate for a non-systematic review format [11].

The PubMed/MEDLINE and Embase databases were searched for relevant articles published through January 2025. The search strategy employed the following terms, used individually and in combination: “non-small cell lung cancer”, “EGFR-mutated non-small cell lung cancer”, “variant allele frequency”, “EGFR tyrosine kinase inhibitors”, “circulating tumor DNA”, “Osimertinib”, and “EGFR tyrosine kinase inhibitor resistance.” The search was limited to studies published in English. Additionally, clinical practice guidelines and relevant publications from major oncology societies were reviewed, including those from the National Comprehensive Cancer Network (NCCN) and the European Society for Medical Oncology (ESMO).

Inclusion criteria encompassed peer-reviewed original research articles, clinical trials, meta-analyses, and practice guidelines addressing EGFR mutation VAF in the context of NSCLC and EGFR TKI therapy. Exclusion criteria comprised case reports with fewer than five patients, conference abstracts without corresponding full-text publications (with noted exceptions for landmark ongoing trials), non-English publications, and studies addressing VAF exclusively in non-EGFR oncogenic drivers.

Titles and abstracts were screened for relevance, followed by full-text assessment of potentially eligible publications. The initial search yielded approximately 320 records. After removal of duplicates and screening of titles and abstracts, 87 full-text articles were assessed for eligibility. A total of 47 studies met the inclusion criteria and were included in the final narrative synthesis. Studies were categorized according to their design (prospective clinical trial, post hoc/exploratory analysis, or retrospective cohort study) to facilitate transparent appraisal of the evidence hierarchy. Evidence was synthesized thematically along the clinical trajectory: baseline risk stratification, on-treatment dynamic monitoring, and resistance characterization. Within each thematic domain, qualitative appraisal was performed considering study design, sample size, consistency of findings across studies, potential sources of bias, and clinical applicability (Table 1).

## 3. The Molecular Landscape of EGFR-Mutated NSCLC and Liquid Biopsy

### 3.1. The Therapeutic Revolution of EGFR Inhibitors and Third-Generation TKIs

NSCLC is the leading cause of cancer mortality worldwide. The emergence of TKIs has fundamentally altered the disease trajectory for patients harboring actionable EGFR mutations; however, the development of acquired resistance remains inevitable. Osimertinib was specifically designed to overcome the most common resistance mechanism to early-generation TKIs—the T790M gatekeeper mutation—while maintaining efficacy against the original activating mutations (primarily exon 19 deletion and L858R) and possessing superior ability to penetrate the central nervous system (CNS). Despite these advantages, tumor heterogeneity and the emergence of complex, often polyclonal, resistance mechanisms necessitate more sensitive and dynamic monitoring tools capable of capturing the evolving molecular landscape of the disease in real time [21,22,23].

### 3.2. Principles of VAF

VAF in liquid biopsy represents the percentage of sequencing reads containing a specific mutation out of the total DNA reads at that genomic locus. Unlike tissue biopsy, which provides a spatially limited snapshot of a single tumor site at a single time point, plasma VAF reflects the aggregate tumor DNA shedding into the bloodstream from all disease sites, thereby offering a more comprehensive portrait of systemic disease biology [24,25].

VAF is influenced by several interconnected biological factors. First, total tumor burden exhibits a strong positive correlation with plasma VAF levels, as larger and more numerous tumor deposits release greater quantities of DNA into the circulation [24,25]. Second, biological activity and necrosis play a role: aggressive tumors with rapid proliferation rates and extensive necrotic areas tend to release larger amounts of ctDNA [24,25]. Third, clonality is a critical determinant—“truncal” mutations present in all tumor cells will generally exhibit higher VAF compared to sub-clonal mutations present only in a fraction of cells, and this metric is central to understanding tumor heterogeneity [24]. Fourth, physiological clearance of DNA fragments by the liver and kidneys affects the steady-state concentration in plasma.

Thus, VAF is not merely a binary metric (“positive/negative”) but a continuous quantitative variable providing rich information on tumor biology. This distinction is fundamental to its clinical utility: rather than simply confirming the presence of a targetable mutation, VAF quantifies the extent to which that mutation dominates the tumor’s molecular architecture [26].

## 4. Baseline VAF Levels: Risk Stratification Prior to Treatment

A fundamental conceptual distinction must be established at the outset: the prognostic interpretation of VAF differs diametrically between tissue-based and plasma-based measurements. High tissue VAF reflects clonal homogeneity of the EGFR-mutant population (tumor purity), indicating critical dependence on EGFR signaling and thus favorable sensitivity to TKI inhibition. Conversely, high plasma VAF primarily reflects elevated systemic tumor burden and aggressive shedding biology, and is associated with inferior outcomes. This inverse relationship is a central theme throughout this review and has important implications for clinical decision-making.

Quantifying EGFR mutation VAF levels in plasma samples obtained prior to osimertinib initiation has demonstrated significant prognostic value across multiple independent studies. High baseline VAF levels are consistently associated with inferior clinical outcomes, serving as a surrogate marker for high tumor burden and aggressive disease biology [27,28,29]. Importantly, the prognostic significance of baseline VAF operates through distinct biological mechanisms depending on the sample source—a nuance that carries critical implications for clinical interpretation.

### 4.1. Correlation Between Tumor Burden and Survival

Multiple studies have sought to quantify the relationship between baseline VAF and survival metrics including progression-free survival (PFS) and overall survival (OS). The convergent finding is that patients with elevated baseline plasma VAF are at increased risk for rapid disease progression, even when treated with a potent agent such as osimertinib.

Quantitative predictive cut-offs have been identified for clinical stratification. One pivotal study established a threshold of 2.6% for the activating EGFR mutation VAF in the second-line setting. Patients with a baseline VAF exceeding 2.6% had a median PFS of only 10 months, compared with a median that was not reached in patients with levels below this threshold (*p* = 0.03). This finding underscores how quantitative differences in circulating tumor DNA translate directly into clinically meaningful disparities in treatment outcomes [26,27].

The prognostic value of VAF is also demonstrable in tissue-based analyses, albeit with an inverse biological interpretation that warrants careful consideration. Research examining adjusted tissue VAF found that high tissue VAF (>70%) predicts longer PFS (52 weeks versus 26 weeks). This apparent paradox relative to plasma findings is explained by the fundamentally different biological phenomena captured by each measurement. High tissue VAF reflects tumor “purity”—a high proportion of tumor cells relative to admixed stromal, immune, and normal epithelial cells—indicating clonal dominance of the EGFR-mutant population. Such clonal homogeneity renders the tumor critically dependent on EGFR signaling and therefore highly susceptible to TKI-mediated inhibition. Conversely, high plasma VAF is primarily a composite measure of total metastatic burden and shedding biology. Factors that elevate plasma VAF include not only tumor volume but also the biological propensity of tumor cells to shed DNA into the circulation, which is influenced by tumor vascularity, necrosis, apoptotic rate, and anatomical location—visceral metastases, for instance, shed substantially more ctDNA than intrathoracic disease alone. Furthermore, high plasma VAF frequently correlates with widespread metastatic dissemination (M1b/M1c staging), which is itself an established independent adverse prognostic factor [30,31]. Critically, tissue VAF is directly influenced by tumor cellularity, defined as the proportion of neoplastic cells relative to the total cell population in the biopsy specimen. Specimens with low tumor cellularity (due to admixed stromal, inflammatory, or normal epithelial cells) will yield lower unadjusted VAF values, potentially leading to underestimation of the true clonal fraction of the EGFR mutation. Pathological assessment of tumor cellularity and mathematical adjustment of VAF for tumor content are therefore essential for accurate interpretation of tissue-based results.

### 4.2. Sensitivity and the “Non-Shedders” Group

A significant subset of patients (approximately 10–20% in trials such as FLAURA and AURA3) are diagnosed with EGFR-positive lung cancer via tissue biopsy but have negative or undetectable ctDNA in plasma. This group, termed “non-shedders,” warrants particular attention [32,33,34].

Data consistently demonstrate that non-shedders enjoy significantly better outcomes. In analyses of the FLAURA and AURA3 trials, patients without detectable baseline ctDNA exhibited superior survival rates. The absence of detectable ctDNA typically indicates lower metastatic burden (M1a versus M1b/c) or intrathoracic-only disease with limited vascular invasion [32,33].

Baseline VAF’s predictive value extends to decisions regarding combination regimens. In the FLAURA2 trial, which evaluated osimertinib plus chemotherapy versus osimertinib monotherapy, the addition of chemotherapy provided the most pronounced benefit to patients with detectable ctDNA at baseline (PFS of 24.8 months with the combination versus 13.9 months with monotherapy). Conversely, for patients with negative baseline ctDNA, the addition of chemotherapy did not significantly improve results (33.3 versus 30.3 months) [34,35]. This differential benefit suggests that baseline ctDNA status could serve as a practical tool for treatment intensity calibration: low VAF identifies a “good prognosis” group adequately treated with osimertinib monotherapy, while high VAF identifies patients who may derive meaningful benefit from treatment intensification.

### 4.3. Genomic Complexity and Co-Mutations

High VAF levels of EGFR mutations are positively correlated with the likelihood of detecting concurrent mutations in tumor suppressor genes such as TP53 and RB1. These co-mutations are known to truncate the duration of treatment response and confer worse prognosis. Notably, however, even in multivariate analyses adjusting for the presence and number of co-mutations, quantitative EGFR VAF retains independent predictive power, indicating that tumor burden itself—as captured by VAF—is a decisive prognostic factor beyond the genomic complexity of the disease [36].

## 5. Dynamic ctDNA Monitoring: Molecular Response and Clearance

Beyond its role in baseline risk stratification, the dynamic behavior of VAF during osimertinib treatment provides a powerful real-time window into molecular response. “Clearance” refers to the decline of VAF levels below the detection threshold of the assay employed. The depth and velocity of this clearance have emerged as among the most robust prognostic indicators in this disease setting.

### 5.1. Clearance Kinetics and Prognostic Significance

Rapid disappearance of mutant alleles from plasma within the first weeks of therapy is strongly linked to durable treatment responses. In a pooled analysis of the FLAURA (first-line) and AURA3 (second-line) trials, ctDNA clearance rates at week 6 were 82% and 65%, respectively. The observed gap between these rates reflects the more complex biology of pre-treated tumors, which harbor greater molecular heterogeneity and may include resistant subclones that are less susceptible to rapid eradication [33].

The predictive power of early clearance is substantial. Patients achieving complete ctDNA clearance by cycle 3 (approximately 9 weeks) demonstrate dramatic improvements in PFS (median 15.2 months versus 6.0 months for non-clearers; HR 0.37, *p* < 0.001) and OS (median 34.0 months versus 17.2 months; HR 0.42, *p* = 0.001). These data position early VAF dynamics as a potentially stronger molecular predictor of long-term benefit than classical clinical characteristics such as performance status or number of metastatic sites [37].

### 5.2. Depth of Reduction vs. Complete Disappearance

A clinically relevant question is whether complete disappearance of ctDNA is required for optimal outcomes, or whether a substantial reduction suffices. While a greater than 50% reduction in VAF is clinically meaningful and associated with improved outcomes compared to non-responders, available data suggest that complete clearance confers a superior prognosis. In the AURA3 trial, although 65% of patients achieved clearance, those with residual measurable ctDNA had intermediate outcomes that were inferior to the clearance group but superior to non-responders, suggesting a graded dose–response relationship between the depth of molecular response and clinical benefit [33,38].

An interesting phenomenon occasionally observed is a temporary sharp spike in ctDNA levels within the first days of treatment—a so-called “tumour flare.” This transient increase is likely attributable to massive tumour cell death releasing DNA into the circulation, followed by a steep decline. Distinguishing this treatment-induced flare from early disease progression requires longitudinal sampling and clinical contextualisation, underscoring the importance of serial rather than single-point VAF assessment [33].

### 5.3. Molecular Progression (mPD)

Longitudinal VAF monitoring enables detection of “molecular progression” (mPD)—the re-emergence of the original mutation or appearance of new resistance mutations—months before progression becomes apparent on conventional imaging (radiological progression, rPD) [36,37,38,39,40,41].

The average lead time between the identification of rising VAF and radiological detection of progression is approximately 2.5 to 6 months [36,37,38,39]. This window potentially affords clinicians an opportunity for early therapeutic intervention. However, current guidelines from ESMO and NCCN do not yet recommend switching treatment based solely on VAF rise in the absence of radiological or clinical evidence of progression, as some patients maintain stable disease for extended periods despite low-level molecular recurrence. Prospective trials evaluating ctDNA-guided treatment switching are required to establish whether acting on mPD confers a survival advantage (Table 2) [40,41].

## 6. The T790M Variable: VAF Ratios and Clonal Architecture (2nd Line)

In the second-line setting, osimertinib efficacy is predicated upon the presence of the T790M resistance mutation. However, the quantitative relationship between T790M VAF and clinical efficacy is considerably more nuanced than a simple binary positive/negative determination.

### 6.1. The T790M/Activating Mutation Ratio

The absolute value of T790M VAF is less informative than its ratio relative to the original activating mutation (e.g., Del19 or L858R). This ratio serves as a proxy for the clonality of the resistance mechanism and, by extension, the degree to which the tumor’s survival depends on T790M-mediated EGFR signaling [4,5,29].

The clonality hypothesis proposes that a high ratio (>0.22–0.30) suggests that T790M is present in the majority of tumor cells driving the disease (truncal resistance), rendering the tumor critically dependent on this pathway and thus highly sensitive to osimertinib. Conversely, a low ratio (<0.22) suggests that only a subpopulation of cells carries T790M (sub-clonal resistance) or that significant molecular heterogeneity exists with alternative resistance mechanisms co-driving disease progression. These patients are at substantially higher risk for rapid progression on osimertinib.

### 6.2. Data Supporting Predictive Thresholds

Research has identified a clinically actionable cut-off for the T790M/activating EGFR mutation ratio at 0.22. Patients with a ratio exceeding 0.22 experienced significantly longer PFS (median not reached versus 6 months, *p* = 0.01) compared to those with a ratio at or below this threshold [4,5,30].

It should be noted that some analyses have found no significant association between T790M abundance and objective response rate or survival. This discrepancy likely stems from methodological heterogeneity, including differences in assay sensitivity and platform (ddPCR versus NGS), variable thresholds for positivity, and differences in patient populations. Nevertheless, the weight of evidence supports the conclusion that, among plasma-positive patients, a high T790M/activating mutation ratio is a favorable prognostic marker [20,30].

### 6.3. Plasma–Tissue Discordance

The source of T790M detection (liquid versus tissue biopsy) fundamentally affects prognostic interpretation. Tissue-positive/plasma-negative patients (“non-shedders”) typically demonstrate better outcomes (PFS approximately 15–16 months) compared to plasma-positive patients, consistent with the principle that undetectable plasma T790M reflects lower systemic disease burden [29]. Among plasma-positive patients, the ratio logic applies; it is prognostically preferable for T790M to be the dominant molecular driver. Importantly, the sensitivity of plasma-based T790M detection is approximately 60–70%, meaning that a negative plasma result does not exclude tissue-based T790M positivity. Patients with negative plasma results must undergo tissue re-biopsy; if tissue confirms T790M, they typically respond well to osimertinib [19,24].

## 7. Genotype-Dependent Dynamics: Exon 19 Deletion vs. L858R

The specific EGFR mutation type exerts a substantial influence on tumor biology, VAF dynamics, and osimertinib efficacy, with implications for clinical management and patient counseling.

### 7.1. Efficacy Gaps

It is well established that patients with exon 19 deletions (Del19) consistently achieve superior outcomes compared to those with L858R point mutations. Median PFS for Del19 is significantly longer (e.g., 21.9 months versus 5.1–12.9 months for L858R) across various clinical trials and real-world datasets [19,31,32,33,34]. At the molecular level, Del19 tumors tend to exhibit deeper and faster VAF clearance. The structural biology of the Del19-mutant receptor permits more efficient kinetic inhibition by osimertinib compared to L858R, leading to more rapid cell death and a sharper VAF decline during treatment [19,35].

### 7.2. Interaction with VAF Levels

High baseline VAF exacerbates the already poorer prognosis of L858R patients. The combination of the L858R genotype, high baseline VAF, and slow clearance constitutes an extremely high-risk molecular profile. In these patients, resistance tends to develop more rapidly, often manifesting with CNS progression patterns [36]. This composite high-risk profile may justify consideration of more intensive upfront therapies, such as osimertinib plus chemotherapy, or implementation of tighter molecular surveillance schedules.

### 7.3. Uncommon Mutations

For uncommon EGFR mutations (e.g., G719X, L861Q, S768I), VAF data remain sparse. Limited available data suggest variable efficacy of osimertinib in these subtypes, making VAF monitoring equally critical—if not more so—for early response assessment and timely identification of treatment failure [37,38].

## 8. Resistance Mechanisms and VAF Evolution

The clinical utility of VAF extends beyond initial response assessment into the detection and molecular characterization of acquired resistance. Longitudinal ctDNA monitoring enables identification of the specific resistance mechanism, which has direct implications for subsequent therapeutic selection.

### 8.1. On-Target vs. Off-Target Resistance

The C797S mutation in EGFR exon 20 represents the most common on-target resistance mechanism to osimertinib. VAF analysis can identify C797S emergence months before clinical failure. Crucially, the allelic configuration of C797S relative to T790M—whether in cis (same allele) or in trans (different alleles)—can be inferred from VAF ratios and dictates sensitivity to combination therapies: trans configurations may retain sensitivity to combined first- and third-generation TKI therapy [39].

MET amplification represents the most frequent off-target bypass mechanism. Detecting MET amplification in cell-free DNA is technically more challenging than detecting point mutations; however, a characteristic ctDNA profile of rising EGFR VAF without emergence of C797S and without on-target mutation clearance often suggests bypass mechanisms such as MET or HER2 amplification, prompting consideration of tissue re-biopsy and MET-directed combination strategies [39].

### 8.2. Dynamics at Progression

A characteristic VAF trajectory during osimertinib therapy has been described: high baseline VAF followed by rapid decline (clearance), a nadir period (undetectable ctDNA), a gradual slow rise (molecular progression), and ultimately an exponential rise coinciding with radiological progression [40,41].

An important observation in the second-line setting is the phenomenon of “loss of T790M” at progression. When the activating mutation VAF rises upon progression but T790M remains low or becomes undetectable, this pattern is associated with shorter subsequent PFS compared to patients who maintain T790M. Loss of T790M suggests a shift in the dominant resistance clone away from EGFR-dependent mechanisms, often toward off-target pathways or histologic transformation [40,41].

### 8.3. Histologic Transformation

Histologic transformation represents a distinct and clinically important resistance mechanism that cannot be reliably detected by liquid biopsy alone. Small cell lung cancer (SCLC) tra nsformation occurs in approximately 3–14% of post-osimertinib resistance cases, while squamous differentiation has also been reported at lower frequencies. The molecular underpinnings of SCLC transformation typically involve concurrent inactivation of RB1 and TP53, which predisposes tumor cells to lineage plasticity and neuroendocrine trans-differentiation [40].

From a VAF perspective, histologic transformation produces a distinctive signature: the original EGFR activating mutation persists at stable or rising VAF (as the transformed cells retain their EGFR mutation), yet no canonical on-target resistance mutations (C797S, T790M loss) are detected in the ctDNA. This “mutation-negative progression” profile—defined as rising EGFR VAF with clinical/radiological progression in the absence of identifiable resistance mutations—should prompt mandatory tissue re-biopsy, as the treatment approach for SCLC-transformed tumors (typically platinum-etoposide chemotherapy) diverges fundamentally from strategies targeting EGFR-dependent resistance [40,42].

### 8.4. Integrative Perspective on Resistance Monitoring

Taken together, the diverse mechanisms of acquired resistance to osimertinib—on-target mutations (C797S), bypass pathway activation (MET, HER2), and histologic transformation—are unified by their detectability through longitudinal VAF monitoring, each producing a distinct and interpretable molecular signature. On-target resistance manifests as the emergence of new mutations within the EGFR gene alongside a rising activating mutation VAF. Bypass resistance is characterized by a rising EGFR VAF without emergence of on-target mutations, often accompanied by detectable copy-number alterations. Histologic transformation produces persistent EGFR VAF with a “mutation-negative” progression profile. This framework positions serial ctDNA-based VAF assessment as a molecular triage tool that, while not replacing tissue biopsy, can guide the urgency and focus of invasive re-sampling and inform initial therapeutic decisions at the time of progression (Table 3).

## 9. Methodological Considerations

The clinical interpretation of VAF levels is inextricably linked to the analytical platform employed, and awareness of methodological limitations is essential for avoiding erroneous conclusions.

### 9.1. Assay Sensitivity: ddPCR vs. NGS

Droplet digital PCR (ddPCR) offers exceptional sensitivity for the detection and quantification of known mutations (e.g., T790M clearance monitoring) but is inherently limited to interrogation of specific pre-defined loci and cannot detect novel or unexpected resistance mutations [40]. Next-generation sequencing (NGS) panels (e.g., Guardant360, Foundation-One Liquid CDx) offer broader genomic coverage, enabling simultaneous detection of co-mutations and emerging resistance drivers. However, NGS sensitivity is generally lower than that of ddPCR, and the “limit of blank” or background noise must be carefully considered when interpreting very low VAF values (<0.5%), as such levels may fall within the assay’s margin of error [38,39,40,41]. The approximate limits of detection (LoD) differ substantially across platforms: ddPCR achieves approximately 0.01–0.1% VAF, NGS panels range from 0.1 to 0.5% for standard methods to approximately 0.01% with unique molecular identifier (UMI)-based error correction, and qPCR-based assays such as the cobas EGFR Mutation Test v2 (Roche Diagnostics) have an LoD of approximately 1–2%. The cobas platform provides a semi-quantitative index (SQI) reflecting the ratio of mutant to wild-type alleles, which, while not directly equivalent to VAF as defined by NGS or ddPCR, has demonstrated clinical utility in monitoring treatment response. For ddPCR, reliable quantification generally requires a minimum of 10,000 total droplets with at least 3 positive droplets for a valid positive call. In NGS, sequencing depth and the use of UMIs for error suppression critically impact the reliability of low-frequency variant detection.

### 9.2. Biological Noise and CHIP

Several commercially available NGS platforms are widely used for ctDNA analysis, including Guardant360 CDx (Guardant Health; 74-gene panel, LoD approximately 0.04–0.1%), Foundation One Liquid CDx (Foundation Medi-cine; 324-gene panel, LoD approximately 0.1–0.5%), and TempusxF (Tempus; 105-gene panel). These platforms employ hybrid-capture enrichment and UMI technology to enhance sensitivity and specificity. It should be noted that RNA-based NGS assays (e.g., Archer Fusion Plex, Oncomine Focus Assay), while valuable for fusion detection and transcript-level variant identification, yield allele frequencies that do not directly correspond to DNA-based VAF due to the influence of transcriptional activity, mRNA stability, and allele-specific expression. Consequently, the evidence base for VAF-based prognostication discussed in this review is built almost exclusively on DNA-based measurements, and RNA-derived allele frequencies should not be used interchangeably with DNA-based VAF for the clinical applications described herein.

Clonal hematopoiesis of indeterminate potential (CHIP) refers to the acquisition of somatic mutations in hematopoietic stem cells that can be detected in cell-free DNA and may confound ctDNA analysis. While EGFR is rarely a CHIP-associated gene, rendering it a highly reliable tumor-specific marker, the analysis of co-mutations—particularly TP53, which is a common CHIP gene—requires careful distinction between tumor-derived and hematopoiesis-derived variants. Paired analysis with matched white blood cell DNA can mitigate this confounding factor [41].

## 10. Clinical Application: Guidelines and Future Directions

### 10.1. Current Guidelines (ESMO/NCCN)

Leading professional bodies acknowledge the clinical utility of ctDNA but remain appropriately cautious regarding real-time treatment modification based on ctDNA data alone. For diagnostic purposes, liquid biopsy is recommended as a “rule-in” test: a positive VAF result for an EGFR activating mutation or T790M confirms the diagnosis and permits treatment initiation, while a negative result is considered inconclusive and necessitates tissue confirmation [42,43,44,45]. Furthermore, the APPLE trial, a randomized phase II study comparing upfront osimertinib versus sequential gefitinib followed by osimertinib upon T790M-mediated progression, provided additional insights into ctDNA monitoring in guiding treatment sequencing. The trial demonstrated higher rates of ctDNA clearance in the upfront osimertinib arm and confirmed that longitudinal ctDNA dynamics can serve as a real-time indicator of treatment efficacy across different therapeutic strategies [46].

Regarding on-treatment monitoring, while ESMO recognizes that VAF changes correlate with clinical outcomes, routine monitoring with the intent to switch therapy based solely on VAF rise (e.g., initiating chemotherapy at molecular progression) is not yet standard of care. The principal barrier is the absence of prospective randomized trials demonstrating that ctDNA-guided early intervention confers a survival advantage over conventional radiological surveillance [40,41].

### 10.2. Future Directions

Several promising avenues for future investigation merit consideration. First, the concept of VAF-guided de-escalation warrants prospective evaluation: patients with deep VAF clearance and low baseline tumor burden may be adequately treated with osimertinib monotherapy, thereby avoiding the toxicity of combination regimens. Available data suggest this subgroup has an excellent prognosis, and prospective de-escalation trials could establish the safety of this approach.

Second, VAF-guided treatment escalation may benefit patients with high-risk molecular profiles (high baseline VAF, slow clearance, L858R genotype, low T790M ratio in the second-line setting), who may be candidates for upfront combination therapies such as osimertinib plus chemotherapy or angiogenesis inhibitors. The FLAURA2 trial has provided initial evidence supporting this approach, demonstrating differential benefit from combination therapy based on baseline ctDNA status [46,47,48,49,50]. Recent data on the addition of anlotinib (an angiogenesis inhibitor) to osimertinib suggest improved PFS in resistant patients, with benefit correlating with the degree of VAF reduction [42,43,44,45].

Third, the extension of VAF monitoring beyond the metastatic setting is gaining momentum. In the adjuvant setting, molecular residual disease (MRD) analyses from the ADAURA trial have demonstrated that ctDNA detection after surgical resection identifies patients at high risk of recurrence, and that osimertinib reduces the rate of ctDNA positivity. These data suggest that the principles of VAF-based monitoring established in advanced disease may be applicable to earlier disease stages [46].

**Figure 1:**
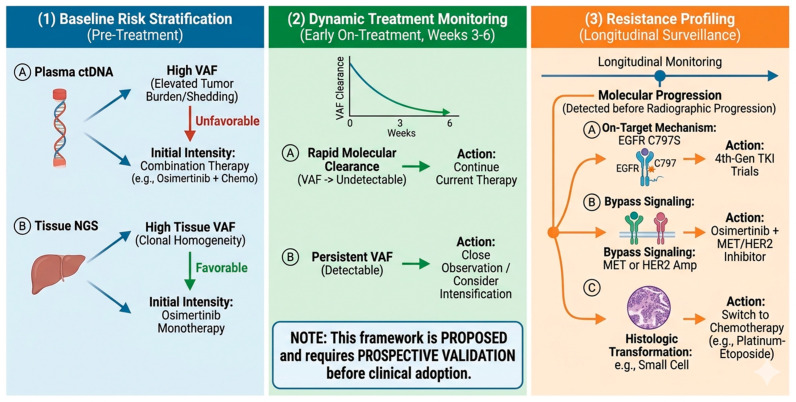
Proposed clinical decision framework for VAF-guided management of osimertinib-treated NSCLC. The framework integrates three sequential dimensions of VAF utility: (**1**) Baseline risk stratification—pre-treatment ctDNA status and VAF levels inform initial treatment intensity (monotherapy vs. combination therapy); (**2**) dynamic treatment monitoring—early on-treatment VAF clearance kinetics (weeks 3–6) provide real-time molecular response assessment; (**3**) resistance profiling—longitudinal VAF surveillance detects molecular progression and identifies specific resistance mechanisms (on-target C797S, bypass MET/HER2 amplification, or histologic transformation), guiding subsequent therapeutic decisions. The figure also illustrates the inverse interpretation of tissue-based VAF (high = favorable, reflecting clonal homogeneity) versus plasma-based VAF (high = unfavorable, reflecting elevated tumor burden). Note: This framework is proposed and requires prospective validation before clinical adoption.

To integrate the evidence discussed throughout this review, we propose a clinical decision framework (Figure 1) that organizes VAF utility along three sequential dimensions: (1) baseline risk stratification using pre-treatment ctDNA status and VAF levels to inform initial treatment intensity; (2) dynamic treatment monitoring using early on-treatment VAF clearance kinetics (weeks 3–6) for real-time molecular response assessment; and (3) resistance profiling using longitudinal VAF surveillance to detect molecular progression, identify specific resistance mechanisms, and guide subsequent therapeutic decisions. This proposed framework requires prospective validation before clinical adoption (Figure 1).

Fourth, ctDNA-guided intervention trials represent an emerging paradigm with the potential to transform clinical practice. The MERMAID-1 and MERMAID-2 trials are evaluating treatment strategies triggered by molecular rather than radiological endpoints, providing a prospective test of the hypothesis that acting on molecular progression can improve survival outcomes. The results of these trials may provide the level of evidence required to shift ctDNA monitoring from a research tool to standard clinical practice [47].

## 11. Discussion

The evidence reviewed herein supports the utility of EGFR mutation VAF as a dynamic, quantitative biomarker spanning the full clinical trajectory of osimertinib-treated NSCLC. However, the strength and maturity of the evidence vary considerably across clinical applications, and several important limitations warrant explicit acknowledgment.

With respect to the quality and consistency of the available evidence, the majority of studies reporting on the prognostic significance of baseline VAF and clearance kinetics derive from exploratory post hoc analyses of randomized controlled trials (e.g., FLAURA, AURA3) or from single-center prospective cohort studies with relatively modest sample sizes. While the direction of effect is remarkably consistent across studies—high baseline VAF predicts inferior outcomes, and early clearance predicts superior outcomes—the specific quantitative thresholds identified (e.g., 2.6% for activating mutation VAF, 0.22 for the T790M ratio) have not been prospectively validated in independent cohorts. This absence of formal validation limits the direct clinical applicability of these cut-offs and underscores the need for standardization efforts.

Sources of inter-study heterogeneity are multiple and merit careful consideration when interpreting the aggregate evidence. These include differences in the analytical platforms employed (ddPCR versus various NGS panels, each with different limits of detection and quantification), variability in the timing of ctDNA sampling (particularly for clearance assessments, which range from week 3 to week 9 across studies), heterogeneity in patient populations (first-line versus second-line, ethnicity, performance status), and inconsistencies in the definition of “clearance” itself (undetectable versus a pre-specified VAF threshold). These factors likely contribute to the discordant findings observed in some analyses, particularly regarding the predictive value of the T790M/activating mutation ratio.

From a conceptual standpoint, the clinical utility of VAF in osimertinib-treated NSCLC can be organized into a three-dimensional framework. The first dimension, baseline risk stratification, employs pre-treatment VAF to categorize patients into prognostic risk groups—from the favorable non-shedder phenotype through intermediate-VAF to high-VAF/high-burden categories—informing initial treatment intensity decisions. The second dimension, dynamic treatment monitoring, leverages early on-treatment VAF kinetics (particularly clearance by weeks 3–6) as a real-time molecular response assessment, potentially enabling adaptive treatment strategies. The third dimension, resistance profiling, utilizes longitudinal VAF surveillance to detect molecular progression, identify the specific resistance mechanism, and guide subsequent therapeutic decisions. The integration of these three dimensions into a unified clinical decision algorithm represents a compelling direction for future research, although prospective validation will be essential.

Several important limitations of the current evidence base should be acknowledged. First, the retrospective or exploratory nature of most analyses introduces potential selection and ascertainment biases. Second, the lack of standardized assay platforms and VAF thresholds across studies limits generalizability. Third, the absence of prospective interventional trials demonstrating that VAF-guided treatment decisions improve overall survival remains the critical barrier to routine clinical implementation. It must be emphasized that no prospective randomized trial has yet demonstrated a survival benefit for treatment modification based on VAF dynamics, and current ESMO and NCCN guidelines do not endorse treatment switching based on ctDNA data alone. Fourth, tissue re-biopsy remains indispensable for certain clinical scenarios—most notably histologic transformation—that cannot be definitively diagnosed by liquid biopsy. Finally, the cost-effectiveness of serial ctDNA monitoring has not been formally evaluated and represents a practical consideration for healthcare systems.

## 12. Conclusions

The impact of VAF levels on osimertinib efficacy in metastatic NSCLC is profound and multidimensional. The synthesis of the reviewed evidence yields the following principal conclusions.

First, high baseline VAF is a negative prognostic marker. Elevated levels of activating EGFR mutations in ctDNA prior to treatment reliably indicate high tumor burden and are independently associated with shorter PFS and OS. Conversely, the absence of baseline ctDNA predicts excellent outcomes and may identify patients suitable for monotherapy approaches.

Second, early ctDNA clearance is the strongest molecular predictor of long-term benefit. The dynamic decline in VAF—specifically the disappearance of measurable ctDNA within the first 3–6 weeks—identifies patients with durable responses. Failure to achieve clearance defines a high-risk group that may benefit from treatment intensification.

Third, in the second-line setting, the T790M/activating mutation ratio matters. Osimertinib efficacy is maximized when the T790M mutation is clonal (ratio > 0.22), whereas low ratios suggest sub-clonal resistance and predict earlier treatment failure.

Fourth, clinical interpretation is methodology-dependent. “Clearance” on a standard assay panel does not equate to total molecular eradication (minimal residual disease negativity), but it nonetheless remains a robust clinical predictor.

For the practicing clinician, VAF provides a “molecular glucometer” for lung cancer (an aspirational analogy reflecting its potential rather than current clinical status)—a dynamic instrument to assess not merely whether the patient harbors the target, but how much of the disease is driven by it and how effectively the treatment is suppressing it in real time. While current guidelines appropriately prioritize radiological assessment for treatment-switching decisions, VAF dynamics offer an early warning system and risk stratification tool that is progressively reshaping precision medicine in EGFR-mutated NSCLC. Prospective trials integrating VAF-guided decision-making into clinical practice are the essential next step toward realizing the full potential of this promising biomarker.

## Figures and Tables

**Table 1:** Summary of key studies on EGFR VAF and osimertinib outcomes included in this review.

Study	Design	*n*	Platform	Setting	Key VAF Finding	Evidence Level
Costa et al. 2024 [12]	RCT (exploratory)	489	NGS	1 L/2 L	ctDNA clearance at week 6: 82% (1 L), 65% (2 L)	Post hoc analysis of phase III RCTs
Del Re et al. 2018/2020 [13]	Prospective cohort	98	ddPCR	2 L	Baseline VAF > 2.6% associated with shorter PFS (10 mo vs. NR)	Single-center prospective
Morikawa et al. 2020 [14]	Multicenter retrospective	318	NGS	Mixed	High tissue VAF (>70%) predicts longer PFS (52 vs. 26 wk)	Multicenter retrospective
FLAURA2 [15,16]	Phase III RCT	586	NGS	1 L	ctDNA+ patients benefit most from combination therapy	Exploratory analysis of phase III RCT
Xie et al. 2026 [17]	Retrospective	186	ddPCR	2 L	T790M/activating mutation ratio > 0.22 predicts longer PFS	Retrospective cohort
Stepien et al. 2023 [18]	Prospective	72	ddPCR	2 L	Clearance by cycle 3: mPFS 15.2 vs. 6.0 mo (HR 0.37)	Single-center prospective
Tsuboi et al. 2021 [19]	Phase III RCT	586	NGS	1 L	Osimertinib +/− chemo; benefit stratified by ctDNA	Phase III RCT
Remon et al. 2023 [20]	Phase II RCT	156	NGS/ddPCR	1 L	Upfront osimertinib: higher ctDNA clearance rates	Phase II RCT (APPLE)

Abbreviations: n, number of patients; RCT, randomized controlled trial; 1 L, first-line; 2 L, second-line; NGS, next-generation sequencing; ddPCR, droplet digital PCR; PFS, progression-free survival; NR, not reached; wk, weeks; mo, months; HR, hazard ratio.

**Table 2:** Impact of ctDNA clearance on survival outcomes in osimertinib-treated NSCLC.

Context (Line)	Study Design	*n*	VAF Checkpoint	Clearance Group	Non-Clearance Group	HR (95% CI)	*p*-Value	Source
General Cohort	Phase II (prospective)	98	Cycle 3	mPFS 15.2 mo	mPFS 6.0 mo	0.37 (0.22–0.63)	<0.001	[29]
FLAURA (1 L)	RCT (exploratory)	279	Week 6	82% clearance	18% non-clearance	N/A	N/A	[38]
AURA3 (2 L)	RCT (exploratory)	210	Week 6	65% clearance	35% non-clearance	N/A	N/A	[41]

Abbreviations: n, number of patients; RCT, randomized controlled trial; mPFS, median progression-free survival; mOS, median overall survival; HR, hazard ratio; CI, confidence interval; mo, months; 1 L, first-line; 2 L, second-line; N/A, not applicable.

**Table 3:** Resistance mechanisms to osimertinib and corresponding VAF dynamics.

Resistance Mechanism	VAF Signature	Frequency	Confirmatory Test	Therapeutic Implication
C797S (on-target, cis with T790M)	Rising EGFR VAF + emergence of C797S; T790M persistent	10–26%	Liquid biopsy (NGS)	No approved TKI effective; clinical trials
C797S (on-target, trans with T790M)	Rising EGFR VAF + C797S on different allele from T790M	Rare	Liquid biopsy; allele-specific analysis	Combination 1st + 3rd gen TKI may be effective
MET amplification (bypass)	Rising EGFR VAF without C797S; no new EGFR mutations	5–22%	Tissue biopsy (FISH/IHC); plasma NGS (CNV)	MET inhibitor combinations (savolitinib, tepotinib)
HER2 amplification (bypass)	Rising EGFR VAF without on-target mutations	2–5%	Tissue biopsy; plasma NGS (CNV)	HER2-directed therapy (T-DXd)
SCLC transformation (histologic)	Persistent EGFR VAF; no resistance mutations detected	3–14%	Mandatory tissue re-biopsy	Platinum-etoposide chemotherapy
Loss of T790M (2 L setting)	Activating mutation VAF rises; T790M undetectable	Variable	Liquid biopsy	Suggests off-target resistance; tissue biopsy recommended

Abbreviations: TKI, tyrosine kinase inhibitor; NGS, next-generation sequencing; FISH, fluorescence in situ hybridization; IHC, immunohistochemistry; CNV, copy number variation; SCLC, small cell lung cancer; 2 L, second-line; T-DXd, trastuzumab deruxtecan.

## Data Availability

The original contributions presented in this study are included in the article. Further inquiries can be directed to the corresponding author(s).

## Abbreviations

The following abbreviations are used in this manuscript:

CHIP	Clonal Hematopoiesis of Indeterminate Potential
CNS	Central Nervous System
ctDNA	Circulating Tumor DNA
ddPCR	Droplet Digital Polymerase Chain Reaction
EGFR	Epidermal Growth Factor Receptor
ESMO	European Society for Medical Oncology
HR	Hazard Ratio
mPD	Molecular Progression
MRD	Minimal Residual Disease
NCCN	National Comprehensive Cancer Network
NGS	Next Generation Sequencing
NR	Not Reached
ORR	Objective Response Rate
OS	Overall Survival
PCR	Polymerase Chain Reaction
PFS	Progression-Free Survival
RB1	Retinoblastoma 1
rPD	Radiological Progression
SANRA	Scale for the Assessment of Narrative Review Articles
SD	Stable Disease
SCLC	Small Cell Lung Cancer
TKI	Tyrosine Kinase Inhibitor
TP53	Tumor Protein p53
VAF	Variant Allele Frequency
